# Association of GII.P16-GII.2 Recombinant Norovirus Strain with Increased Norovirus Outbreaks, Guangdong, China, 2016

**DOI:** 10.3201/eid2307.170333

**Published:** 2017-07

**Authors:** Jing Lu, Ling Fang, Limei Sun, Hanri Zeng, Yanling Li, Huanying Zheng, Siwei Wu, Feng Yang, Tie Song, Jinyan Lin, Changwen Ke, Yonghui Zhang, Jan Vinjé, Hui Li

**Affiliations:** Guangdong Provincial Center for Disease Control and Prevention, Guangzhou, China (J. Lu, L. Fang, L. Sun, H. Zeng, Y. Li, H. Zheng, S. Wu, F. Yang, T. Song, J. Lin, C. Ke, Y. Zhang, H. Li);; Centers for Disease Control and Prevention, Atlanta, Georgia, USA (J. Vinjé)

**Keywords:** Norovirus GII.2 genotype, viruses, gastrointestinal illness, Guangdong, China, foodborne disease, enteric infections, food safety, norovirus

## Abstract

An unusual prevalence of recombinant GII.2 noroviruses (GII.P16-GII.2) in Guangdong, China, at the end of 2016 caused a sharp increase in outbreaks of acute gastroenteritis. This event was another non-GII.4 epidemic that emerged after the GII.17 viruses in 2014 and 2015 and warrants global surveillance.

During the past 20 years, GII.4 genotypes have been responsible for most norovirus outbreaks globally (*1*). New GII.4 variants have emerged every 2 or 3 years, replacing the previous dominant variant. Likewise, in Guangdong, China, as well as in other regions of China, GII.4 noroviruses have caused most outbreaks; several other genotypes, such as GI.3, GI.6, GII.6, and GII.21, were occasionally detected in sporadic cases but rarely caused large outbreaks (*2*).

The Guangdong provincial surveillance network for foodborne disease outbreaks has been active since 2008 and is responsible for the surveillance of norovirus outbreaks in Guangdong. During winter 2014–15, a new GII.17 variant (GII.P17-GII.17 Kawasaki) was first identified in Guangdong and caused a substantial increase in the number of acute gastroenteritis outbreaks (*3*). Epidemics caused by this lineage were detected almost simultaneously in several other provinces of China, and sporadic cases were reported worldwide (*4*). We report an increase in the number of outbreaks associated with a GII.P16-GII.2 recombinant norovirus strain in the last months of 2016 in Guangdong.

## The Study

**Figure 1:**
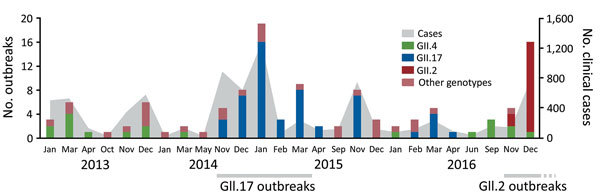
Number of reported GII norovirus outbreaks and confirmed clinical cases, Guangdong, China, January–December 2016.

In Guangdong, norovirus outbreaks are highly seasonal; most (>90%) are reported during November–March (*3*). In November 2014, a GII.17 variant was identified in Guangdong and became predominant during the 2014–15 and 2015–16 norovirus seasons (*5*). After June 2016, the number of GII.17 outbreaks decreased, replaced by GII.4 outbreaks (Figure 1). During November and December 2016, a sharp increase in the number of norovirus outbreaks was reported in multiple cities of Guangdong through the provincial surveillance network (Figure 1). Seventeen (81%) of the 21 outbreaks in 10 cities were typed as GII.2; these outbreaks resulted in 760 clinical cases (Figure 1). This genotype was first detected in Guangzhou on November 14, 2016, and spread rapidly thereafter. The sharp increase in the number of outbreaks and the unusual GII.2 genotype prompted us to further characterize these viruses.

**Figure 2:**
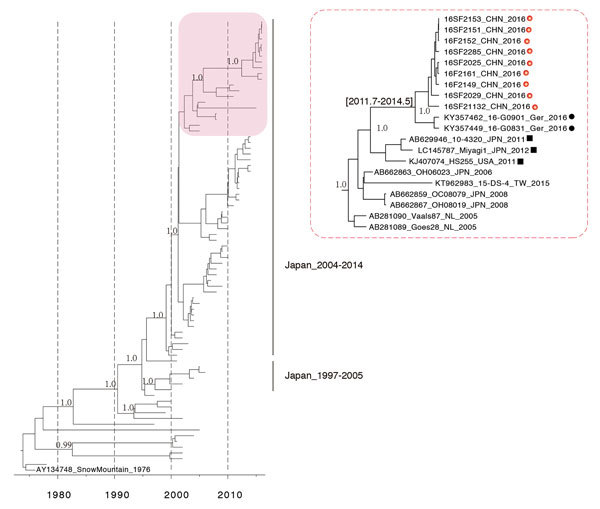
Molecular clock phylogeny of norovirus strain GII.2 VP1 gene sequences. The tree is a maximum clade credibility phylogeny with the GII.2 VP1 sequences, including the Guangdong, China, outbreak strains (red box, enlarged at right). Red dots indicate GII.2/Guangdong/2016 strains; black dots indicate outbreak strains from Germany, 2016; black squares indicate closely related GII.2 strains reported in previous years.

We randomly selected 5 fecal samples from each GII.2 outbreak for Sanger sequencing. The full length of *RdRp* and *VP1* genes were sequenced by using specific oligonucleotide primer sets (Technical Appendix Table) Representative sequences were deposited in GenBank (accession nos. KY485107–KY485126). A primer set (JV12RY and JV13I [*6*]) commonly used for RdRp typing failed to amplify the corresponding fragments of GII.2/Guangdong/2016 strains because of mismatches with these primers. We compared the Guangdong GII.2 viruses with related *VP1* (GII.2) and *RdRp* gene sequences (P16) from GenBank. Molecular clock phylogenetic analysis was performed to analyze the evolution of the *VP1* gene sequences by using the Bayesian Markov chain Monte Carlo framework with a generalized time-reversible nucleotide substitution model (*7*) and an uncorrelated lognormal relaxed clock model (*8*) (Figure 2). Most GII.2 sequences before 2004 uploaded to GenBank were from Japan, the United States, and the Netherlands (Technical Appendix Figure 1). GII.2 strains detected after 2004 clustered into a major lineage that included the viruses detected in Guangdong in 2016. This lineage, which also included the GII.2 viruses reported in Germany (*9*), was estimated to have emerged during 2011–2014 (95% highest posterior density 2011.7–2014.5) and was divergent from GII.2 strains detected in Japan in 2014. The genetically closest *VP1* gene sequences were GII.2 viruses from the United States in 2011 and from Japan in 2011 and 2012 (Figure 2). Consistent with the tight cluster of the capsid sequences, the GII.P16 *RdRp* gene sequences of the Guangdong and Germany outbreaks were closely related with GII.P16 sequences from GII.4 Sydney viruses detected in Japan and the United States (Technical Appendix Figure 2).

## Conclusions

We report an increased number of norovirus GII.2 outbreaks during fall 2016 in Guangdong Province, China. The provincial surveillance network reported 17 GII.2 norovirus outbreaks that affected 760 persons during November 14–December 28, 2016. On the basis of complete viral polymerase gene (*RdRp*) and capsid gene (*VP1*) nucleotide sequences, all viruses could be typed as GII.P16-GII.2.

The driving force behind the emergence and spread of GII.2 noroviruses in Guangdong Province is not clear, but the fact that all viruses had a GII.P16 *RdRp* gene might indicate that possession of this polymerase gene makes these viruses more virulent. Similar GII.P16 *RdRp* genes have been reported as part of GII.4 Sydney viruses recently identified in Japan (GenBank accession no. LC153122, GII.P16-GII.4/JP/2016) (*10*) and the United States (GenBank accession no. KX907727, GII.P16-GII.4/U.S./2015) (Technical Appendix Figure 1), which suggests that recent recombination events of GII.2 and GII.4 Sydney viruses with GII.P16 *RdRp* gene sequences might have been responsible for most of the recent norovirus activity.

The number of GII.P16-GII.2 outbreaks peaked at the end of 2016, similar to the epidemic season caused by the GII.P17-GII.17 variant in 2014. The emergence of GII.2 viruses was almost simultaneously reported in several other regions of China (China National Surveillance Network for Foodborne Disease Outbreaks, February 2017) as well as in Germany (*9*), suggesting the fast spread of this genotype on multiple continents. As a result, the predominant norovirus genotype in Guangdong Province has changed from GII.4 viruses, of which a new variant emerged every 2 or 3 years, to non-GII.4 viruses with GII.17 during the 2014–15 winter season and GII.2 viruses during the 2016–17 season. Since the GII.17 variant has spread to several countries in Asia and has been reported on different continents, the effect of this emerging GII.2 recombinant strain warrants further global surveillance.

Technical Appendix Primer sets used to amplify and sequence norovirus *RdRp* and *VP1* genes; molecular clock phylogeny of GII.2 VP1 gene sequences; maximum-likelihood trees for RdRp gene.
