# Supplementary material for: Association of GII.P16-GII.2 Recombinant Norovirus Strain with Increased Norovirus Outbreaks, Guangdong, China, 2016
Source: Emerg Infect Dis. 2017 Jul;23(7):1188–90. doi: 10.3201/eid2307.170333 (PMC5512473; doi:10.3201/eid2307.170333)
Supplement: Technical Appendix — Primer sets used to amplify and sequence norovirus RdRp and VP1 genes; molecular clock phylogeny of GII.2 VP1 gene sequences; maximum-likelihood trees for RdRp gene. [file 17-0333-Techapp-s1.pdf]

Article DOI: https://dx.doi.org/10.3201/eid2307.170333

Association of GII.P16-GII.2 Recombinant Norovirus Strain with Increased Norovirus Outbreaks, Guangdong, China, 2016

Technical Appendix

Technical Appendix Table. Primer sets used to amplify and sequence norovirus *RdRp* and *VP1* genes, Guangdong, China, 2016

Primer	Sequences (5′→3′)
NV_P16_RdRp-forward	YCTTCTRCGCCCATTYC
NV_P16_RdRp-reverse	YCTTCTRCGCCCATTYC
GII.2_VP1_1-forward	GAATGAAGATGGCGTCGAATG
GII.2_VP1_1- reverse	TTRAAWGCRCAAATRCCACTRAC
GII.2_VP1_2-forward	TCYAATTCHAGRTTYCCAGTG
GII.2_VP1_2-rReverse	YCTTCTRCGCCCATTYC

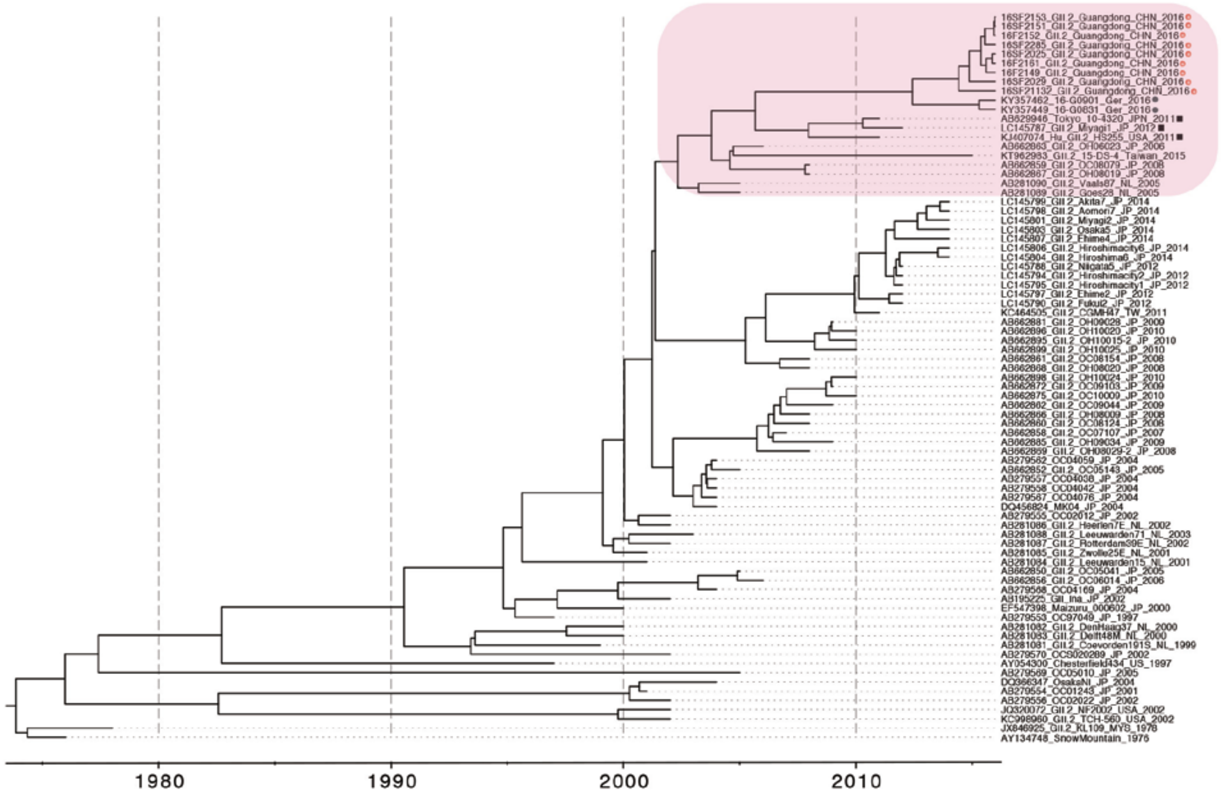

Technical Appendix Figure 1. Molecular clock phylogeny of norovirus GII.2 VP1 gene sequences with GenBank accession numbers and regions. Red box indicates GII.2 VP1 sequences, including the Guangdong, China, outbreak strains. Red dots indicate GII.2/Guangdong/2016 strains; black dots indicate outbreak strains from Germany, 2016; black squares indicate closely related GII.2 strains reported in previous years.

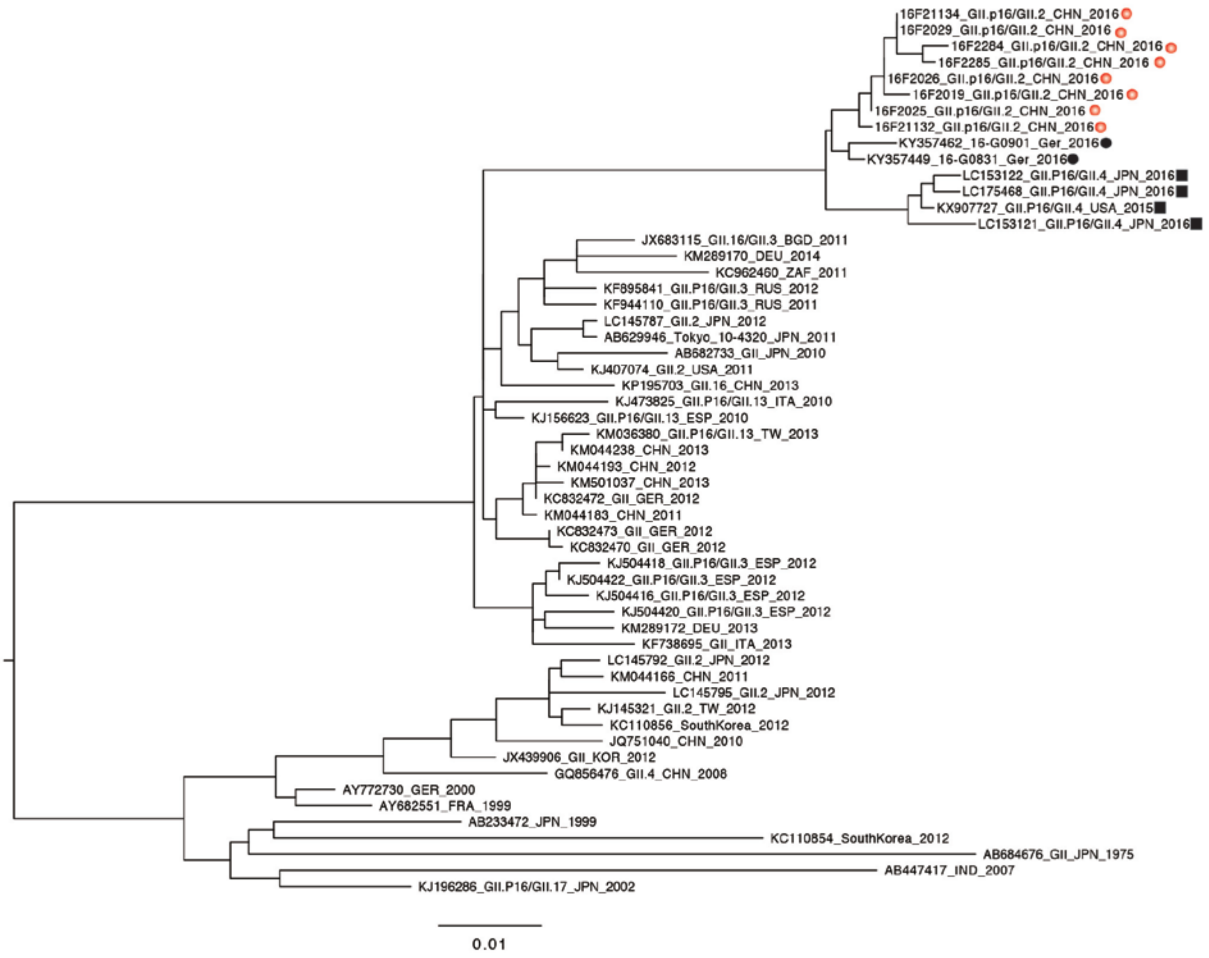

Technical Appendix Figure 2. Maximum-likelihood trees for norovirus RdRp gene in RaxML (*1*) using the generalized time-reversible with γ-distributed rates among sites. Black squares indicate GII.4 viruses with a GII.P16 gene similar to the GII.P16 gene of the GII.2/Guangdong/2016 strains. Scale bar indicates nucleotide substitutions per site.

Reference

1. Stamatakis A. RAxML-VI-HPC: maximum likelihood-based phylogenetic analyses with thousands of taxa and mixed models. Bioinformatics. 2006;22:2688–-90. **PMID: 16928733**
